# Project: Otorhinolaryngology Trails

**DOI:** 10.1590/S1808-86942012000400001

**Published:** 2015-10-20

**Authors:** Prof. Dr. Marcelo Hueb

**Affiliations:** Chairman of the Brazilian Association of Otorhinolaryngology and Facial and Neck Surgery

The pleasant invitation and, consequently, the task of writing an editorial about the Caminhos da Otorrinolaringologia (Otorhinolaryngology Trails) campaign for a scientific journalseemed to me odd and illogical at the time. Notwithstanding, upon pondering on what is happening, I realized it did have a lot which certainly fit in the scientific realm. The invitation was not by change, since all of this can be compared to a major scientific project!

The background certainly sprang from my participation in numerous initiatives from the “supra-specialties” (Voice Week, Hearing Health, Breathe Through Your Nose and Live Better) in the local or regional coordination team; also coordinating the state-wide Voice Week campaign in Minas Gerais, in 2003. The national reach of this “training” peaked when I participated in the National Coordination of the Hearing Health Campaign, in 2007. With such foundation, I devised the campaign with two major and essential goals in mind: Education in Anatomo-Physiology and Prevention in Otorhinolaryngology and bringing our specialty closer to the public.

Upon devising this prospective and longitudinal contemporary cohort project, I went ahead to execute it. After meetings with the Executive Directors and having the approval from the Ethics Committee (Chairs of the “Supra-Specialties”), we received funds from the Fiscal and Administrative Council of the Brazilian Association of Otorhinolaryngology and Facial and Neck Surgery - ABORL-CCF. Having everything properly outlined, but still with a strong need to reach the public and make our national campaign official, we orchestrated an important support from the press and the Ministry of Health.

**Figure 1:**
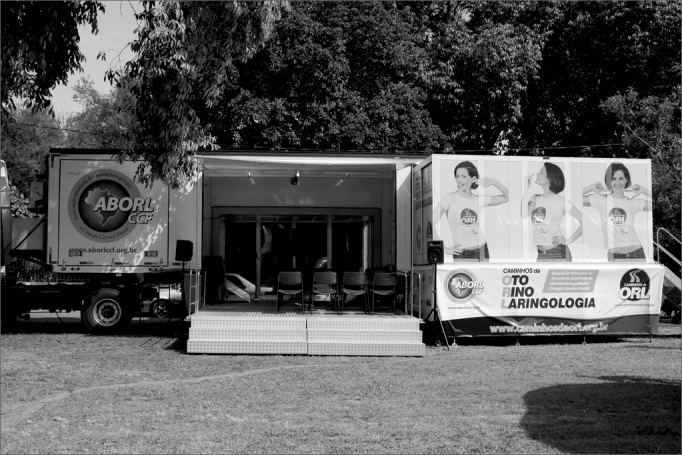
Open mobile unit with the miniauditorium.

**Figure 2:**
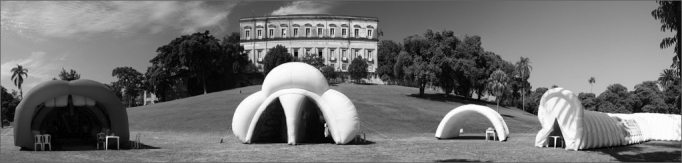
Panoramic view of the ear, nose and through inflatable anatomical models.

We then set off to the field activities, which will encompassed 17 cities in 12 Brazilian states and the Federal District. We have a 17-meter long trailer, fully converted for the campaign, with 3 different compartments (polysomnography room, an auditorium with audio-visual system and a press/living room). We also have gigantic inflatable anatomical models representing the ear, nose and mouth/larynx (Figures 1 and 2). These models enable the person to literally “set foot in” the organs, seeing details of anatomy and physiology, while also receiving information on disease prevention.

The preliminary discussion and analysis or our results have shown that the longitudinal prospective outline have marked cross-sectional traits, with branding facts and moments in each one of the stages/cities where we deployed your campaign, leaving good memories and friendship and, thus, having a historical retrospective cohort view. The conclusions from such journey matched the proposed objectives, adding social responsibility and awareness about our association and our specialty. I hope this Project can become a “line of research” for “future researchers” within ABORL-CCF.

Best Regards!

